# Exploring Access to Surgical Interventions for Hidradenitis Suppurativa: Retrospective Population-Based Analysis

**DOI:** 10.2196/31047

**Published:** 2021-12-14

**Authors:** Alexandra Finstad, Alex Lee, Ralph George, Raed Alhusayen

**Affiliations:** 1 Faculty of Medicine University of Ottawa Ottawa, ON Canada; 2 Division of General Surgery Department of Surgery University of Toronto Toronto, ON Canada; 3 Department of Surgery Canadian Imperial Bank of Commerce Breast Centre St. Michael’s Hospital Toronto, ON Canada; 4 Division of Dermatology Department of Medicine University of Toronto Toronto, ON Canada; 5 Division of Dermatology Department of Medicine Sunnybrook Health Sciences Centre Toronto, ON Canada

**Keywords:** hidradenitis suppurativa, surgery, dermatology, access, epidemiology, universal health care

## Abstract

**Background:**

Hidradenitis suppurativa (HS) is a painful inflammatory disorder that confers significant distress to patients, with surgery as an integral treatment modality.

**Objective:**

To inform improvements in care, patterns in HS surgery were assessed.

**Methods:**

A retrospective population-based analysis was performed on Ontario billing claims for HS surgery across a period of 10 years from January 1, 2008 to December 31, 2017. HS surgery was defined as the excision of inguinal, perineal, or axillary skin and sweat glands for hidradenitis. The top 5 billing specialties, including general and plastic surgery, were analyzed. The total number of procedures performed as well as the number performed per physician were investigated. Patient and physician locations were compared.

**Results:**

A total of 7195 claims for the excision of inguinal, perineal, or axillary skin and sweat glands for HS were submitted across the study period. Annual HS surgery claims showed an increasing trend across 10 years, ranging between 4.9 and 5.8 per 100,000 population. However, overall, for every additional year, the number of claims per 100,000 population only increased slightly, by 0.03 claims. The number of providers steadily decreased, ranging between 1.7 and 1.9 per 100,000, with approximately twice as many general than plastic surgeons. However, again overall, for every additional year, the number of providers per 100,000 population decreased slightly, by 0.002 physicians. The mean annual number of procedures per physician rose from 2.8 to 3.1. In rural areas, analyzed per claim, general surgeons performed the majority of surgeries (1318/2003, 65.8%), while in urban areas, surgeries were more equally performed by general (2616/5192, 50.4%) and plastic (2495/5192, 48.1%) surgeons. Of HS surgery claims, 25.7%-35.9% were provided by a physician residing in a different area than the patient receiving care.

**Conclusions:**

No significant improvements in access to HS surgery were seen across the study period, with access potentially worsening with annual HS claims rising overall and number of providers decreasing, with patients travelling further to access surgery. System barriers across the continuum of HS diagnosis and management must be evaluated to improve access to surgical care.

## Introduction

Hidradenitis suppurativa (HS) is a painful, inflammatory disorder involving a dysfunction of the pilosebaceous unit, which confers significant distress to patients due to its relapsing and remitting nature [1-3]. ﻿HS management is complex, with both medical and surgical treatment options and the first North American clinical management guidelines only recently published in 2019 [4,5]. A variety of options for medical treatment is available depending on the severity of disease, including topical and intralesional therapies, systemic antibiotics, hormonal therapies, retinoids, immunosuppressants, and biologics [4,5]. However, surgery remains an integral treatment modality regardless of disease severity [4-6]. Approximately 80% of patients were shown to be satisfied with surgical treatment of HS and considered it as the best treatment option [6].

Unfortunately, the diagnosis of HS is relatively rare, and it is often mistaken for a simple infection, limiting access to proper treatment [7]. HS is commonly diagnosed after a significant delay, with one multinational study reporting a mean delay of 10.2 years [8] and Canadian data reporting a median delay of 7 years with an average of 3 misdiagnoses [9]. Treatment has also been found to be fragmented over multiple specialties, including dermatology, primary care, general surgery, and plastic surgery [10,11], with ﻿patients trying an average of 15 different methods to manage their HS symptoms [9]. The absence of a designated specialty for HS management has been suggested to further delay diagnosis and treatment [12,13].

The prevalence of HS has been reported to range from 0.03% to 4.10% [14]. Although guidelines have been published for the surgical management of HS, they commonly rely on low-quality, uncontrolled, retrospective reports, and whether there is adequate access to HS surgery is unknown [5]. Moreover, although early surgical interventions are believed to potentially prevent progression of disease, data are sparse, and the extent of adoption of surgical management for HS is unclear [15].

To inform improvements in HS care, patterns in current and past HS surgery must be assessed. The objectives of this study were to evaluate patient access to surgical procedures for HS and investigate trends in HS surgery across different specialties and geographical regions.

## Methods

### Data Source

Ontario was chosen as the study setting as it is the most populous province in Canada with approximately 14.7 million inhabitants [16] and provides its citizens with universal health care through the Ontario Health Insurance Plan (OHIP). Data on OHIP medical claims were obtained from the Medical Services and Population data source within IntelliHealth, a province-wide data repository operated by the Ontario Ministry of Health and Long-Term Care containing information on physician billing. IntelliHealth has been utilized in prior population-based studies on physician billing and practices [17-22]. Research ethics board approval was not required for this study as information obtained through IntelliHealth is anonymized and publicly available.

### Study Population

A retrospective, population-based analysis was performed on Ontario physicians who surgically treated HS. Data were collected across 10 years from January 1, 2008 to December 31, 2017. Physicians who surgically treated HS or hyperhidrosis were identified by procedure codes R059 (unilateral excision of inguinal, perineal, or axillary skin and sweat glands for hyperhidrosis and/or hidradenitis) and R060 (excision of inguinal, perineal, or axillary skin and sweat glands for hyperhidrosis and/or hidradenitis with skin graft(s) or rotation flap(s)) [23]. From these, claims billed under diagnostic code 799 for “excessive sweating” (hyperhidrosis) were excluded to further isolate those for HS, as no OHIP diagnostic code currently exists for HS [24]. The top 5 billing specialties were analyzed, excluding family physicians and anaesthesiologists, to further ensure that the procedure was being performed for the purposes of HS.

### Data Analysis

Data were exported from IntelliHealth’s online system and analyzed using Microsoft Excel version 16.36. Physician specialty was defined as the specialty billed for the procedure. The number, location, and specialty of physicians who performed the excision of inguinal, perineal, or axillary skin and sweat glands for HS were analyzed. The total number of procedures performed as well as the number performed per physician were investigated. Patient and physician locations were compared. Location was determined based on the assigned Local Health Integration Network (LHIN). Each LHIN was further classified as rural or urban following previously applied methodology in which a LHIN is deemed rural if its population is less than 1,000,000 and urban if greater [17,20,21].

## Results

### Demographics

**Figure 1:**
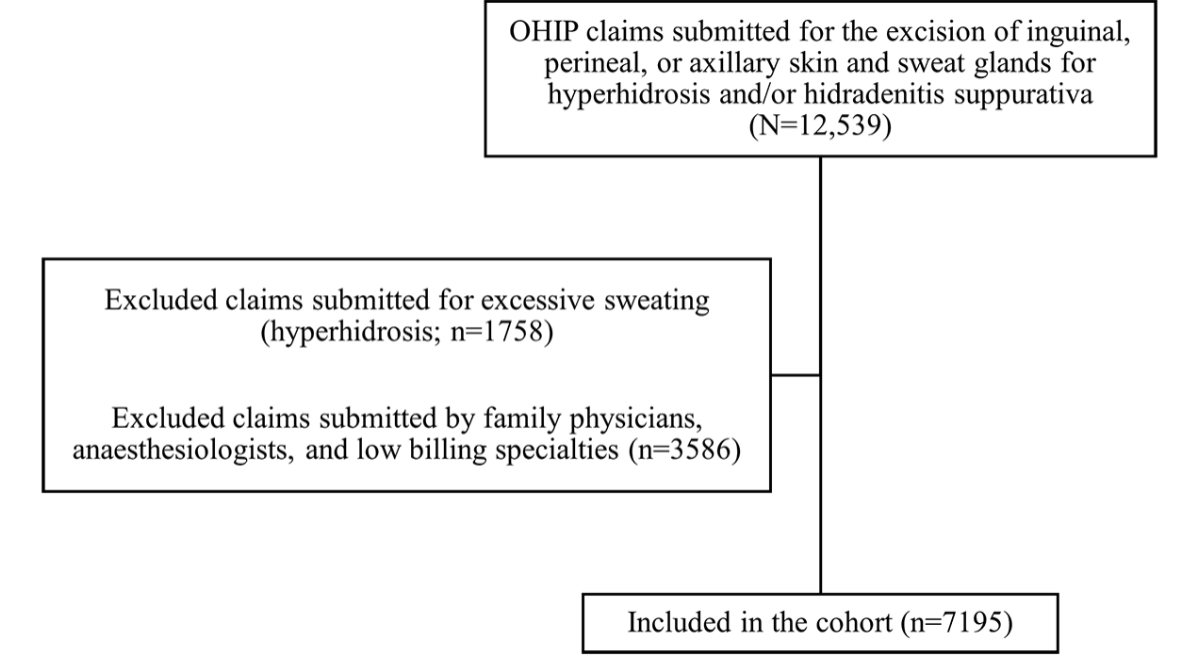
Cohort formation flowchart. OHIP: Ontario Health Insurance Plan.

Across the study period, a total of 12,539 claims were submitted for the excision of inguinal, perineal, or axillary skin and sweat glands for hyperhidrosis and/or HS. Of these cases, 1758 were excluded because they were submitted for hyperhidrosis (excessive sweating). A further 3586 claims were excluded based on specialty billed. A final total of 7195 claims was included in the study (Figure 1). Patient demographics are shown in Table 1 for patients with a valid health card number. Approximately 10% of patients had multiple surgeries over the study period.

**Table 1:** Ontario hidradenitis suppurativa surgery patient demographics.

Characteristics		2008 (n=569)	2009 (n=628)	2010 (n=624)	2011 (n=631)	2012 (n=704)	2013 (n=667)	2014 (n=669)	2015 (n=640)	2016 (n=668)	2017 (n=670)
**Sex, n (%)**											
	Female	366 (64.3)	427 (68.0)	441 (70.7)	433 (68.6)	458 (65.1)	453 (67.9)	454 (67.9)	415 (64.8)	426 (63.8)	419 (62.5)
	Male	203 (35.7)	201 (32.0)	183 (29.3)	198 (31.4)	246 (34.9)	214 (32.1)	215 (32.1)	225 (35.2)	242 (36.2)	251 (37.5)
Age (years), mean		40.4	40.8	41.0	40.7	40.2	40.3	39.9	41.8	41.6	42.8
**Age (years), n (%)**											
	0-19	41 (7.2)	26 (4.1)	31 (5.0)	36 (5.7)	55 (7.8)	36 (5.4)	32 (4.8)	30 (4.7)	30 (4.5)	31 (4.6)
	20-44	306 (53.8)	350 (55.7)	349 (55.9)	351 (55.6)	386 (54.8)	372 (55.8)	396 (59.2)	347 (54.2)	381 (57.0)	340 (50.7)
	45-64	181 (31.8)	215 (34.2)	203 (32.5)	194 (30.7)	216 (30.7)	217 (32.5)	192 (28.7)	219 (34.2)	197 (29.5)	234 (34.9)
	65-74	23 (4.0)	24 (3.8)	25 (4.0)	36 (5.7)	29 (4.1)	29 (4.3)	36 (5.4)	28 (4.4)	35 (5.2)	46 (6.9)
	≥75	18 (3.2)	13 (2.1)	16 (2.6)	14 (2.2)	18 (2.6)	13 (1.9)	13 (1.9)	16 (2.5)	25 (3.7)	19 (2.8)

### Providers of HS Surgery

**Figure 2:**
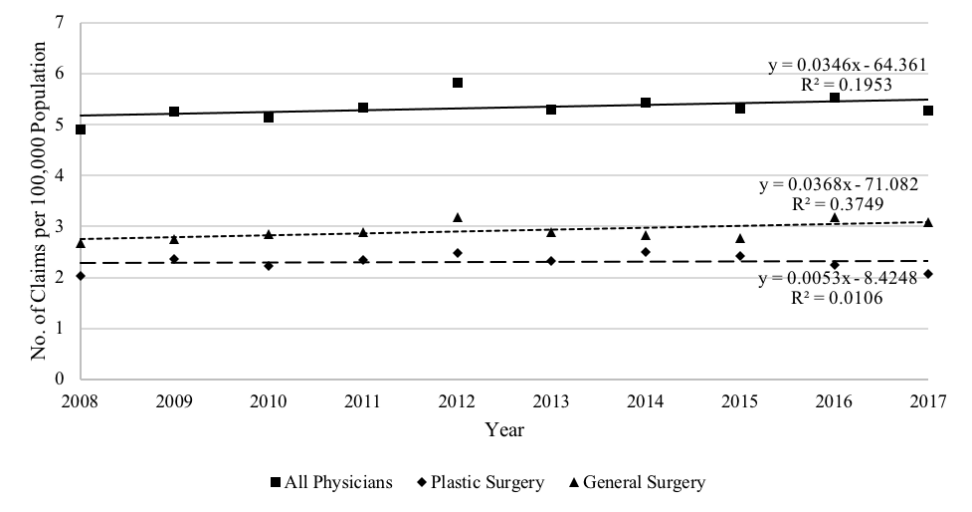
Submission of claims for hidradenitis suppurativa surgery over time per 100,000 population.

The top 5 billing specialties for HS surgery were general surgery, plastic surgery, obstetrics and gynecology, urology, and dermatology (Multimedia Appendix 1, Supplemental Table 1). Over the course of the entire study period, general and plastic surgeons submitted the vast majority of claims for surgical treatment of HS, at 3934 and 3107 claims, respectively. General surgeons performed the majority of unilateral excision procedures (R059), while plastic surgeons performed the majority of procedures that involved a skin graft or rotation flap (R060). The annual number of claims submitted for HS surgery experienced an overall slight increase across the study period when standardized by population, ranging between 4.9 (2008) and 5.8 (2012) claims per 100,000 population (Figure 2). However, overall, for every additional year, the number of claims per 100,000 population only slightly increased, by 0.03 claims.

**Figure 3:**
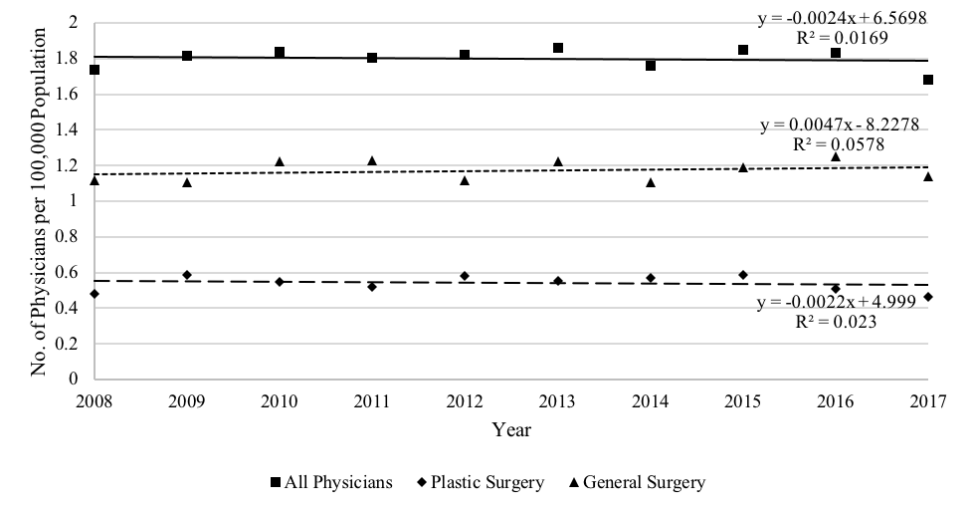
Providers of hidradenitis suppurativa surgery over time per 100,000 population.

By specialty, annual claims submitted by general surgeons increased slightly more than those by plastic surgeons. The number of HS surgery providers per 100,000 population ranged from 1.7 to 1.9, with general surgeons ranging from 1.1 to 1.3 and plastic surgeons ranging from 0.5 to 0.6 (Figure 3). However, overall, for every additional year, the number of providers per 100,000 population decreased slightly, by 0.002 physicians.

**Figure 4:**
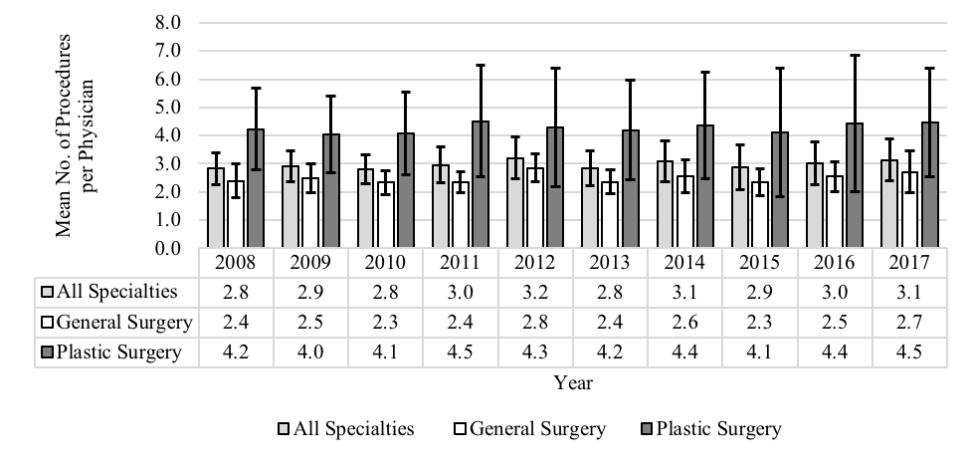
Annual hidradenitis suppurativa surgeries performed per physician.

The mean number of procedures performed annually per physician rose from 2.8 to 3.1 across 10 years (Figure 4). Plastic surgeons performed more procedures per physician than did general surgeons, ranging from 4.0 to 4.5 compared with 2.3 to 2.8. However, the change in procedures per physician over time for all providers, as well as plastic surgeons and general surgeons, was not statistically significant. It is also important to note that, averaged across the 10 years, 85.2% (2069/2427) of physicians submitted <5 claims per year, while only 14.8% (358/2427) submitted ≥5 claims per year. Furthermore, many only performed 1 HS surgery per year, therefore not necessarily qualifying as a specialized provider of HS care.

### Geographic Distribution of Claims

In total, analyzed per claim, more patients (2281/7195, 31.7%) than physicians (2003/7195, 27.8%) resided in a rural area, while more physicians (5192/7195, 72.2%) than patients (4914/7195, 68.3%) resided in an urban area (see Multimedia Appendix 2, Supplemental Figure 1a). Furthermore, over time, HS surgeries were increasingly being performed by physicians residing in a different geographic region than the patient receiving care (low of 176/684, 25.7% in 2009 to a high of 269/749, 35.9% in 2017; Multimedia Appendix 3, Supplemental Figure 2). When comparing surgeries performed in rural areas to those in urban areas, in rural areas, surgery was most often performed by a general surgeon (1318/2003, 65.8%), while in urban areas, surgeries were more equally performed by general surgeons (2616/5192, 50.4%) and plastic surgeons (2495/5192, 48.1%; Multimedia Appendix 2, Supplemental Figure 1b).

## Discussion

### Principal Findings

In this population-based analysis of HS surgical care in Canada, there was a slight trend towards increasing number of claims for HS surgery per 100,000 population over the 10-year study period, while the number of providers per 100,000 population decreased, particularly in general surgery. However, procedures performed per physician increased overall, although the increase was not statistically significant. Geographically, patients were also travelling further to access surgery. These findings suggest that overall access to HS surgery has not significantly improved over the study period and in fact may be decreasing as more and more patients seek care away from home as the number of clinicians providing surgery decreases.

The female:male ratio of patients included in this study was similar to that of a previous report on surgical interventions for HS patients in Ontario [25]. In comparison, the general female:male ratio among all HS patients is 3:1 [26-28], suggesting that men diagnosed with HS are more likely to undergo surgery than women. This is possibly due to men generally experiencing more severe disease [28,29], underestimation and dismissal of pain in female patients [30], or a lack of surgical expertise for female care due to common lesion localization to the inguinofemoral area [28], as well as low numbers of gynecologists providing HS surgery. This emphasizes the need for improved access to care for female patients through improved surgical expertise and increased awareness surrounding perception of pain in women.

The mean age of disease onset has been reported as 20.5 (SD 9.3) years, with a mean age at diagnosis of 30.7 (SD 10.9) years, representing a mean delay from onset of symptoms to diagnosis of 10.2 (SD 8.9) years [8]. In our study, the mean age at the time of surgery was in the early 40s and increased by 2 years during the study period, representing a duration of potentially 10 more years from diagnosis to surgical treatment. One reason for the general rise in the mean age of patients undergoing HS surgery and the decrease in claims among patients 20-44 years old may be that medical therapy for control of early disease is increasingly being utilized over surgery, which remains a necessary adjunct intervention for refractory HS cases [6,10,31,32]. The mean age of surgery may also be increasing as patients may be receiving multiple surgeries, found to be approximately 10% in this study, with surgical intervention potentially starting at a later age due to surgical options only being explored once the disease has progressed to a more severe state. Furthermore, in Canada, 3.8% of the population, or approximately 3800 per 100,000 persons, are estimated to be living with HS [33]. Exact estimates of HS severity still vary widely, with reports of 3.9% to 23.7% of HS patients diagnosed with Hurley Stage III [28,29,34]. However, in this study, only 4.9 to 5.8 claims per 100,000 persons were submitted annually for the surgical excision of HS. Despite the considerable number of severe cases reported in the literature, surgical management of HS may therefore be underutilized.

Other recent advances in HS care have focused on new systemic drugs that target different immune mediators in the pathogenesis of HS [35]. Research on various monoclonal antibodies and small molecules are currently underway, while the use of anti-tumor necrosis factor biologic therapy has already demonstrated reductions in HS severity in clinical trials [35]. However, patients have reported high satisfaction with surgical management and experience relatively low recurrence of HS [5,6,36,37]. This suggests the need to consider surgery earlier as part of HS management to limit the long-term morbidity and prolonged progression of the disease. Combined management with biologic therapy has also been advocated in the setting of moderate-to-severe disease [38].

HS surgery was found to be primarily performed by general and plastic surgeons, consistent with previous literature [13]. Although general surgeons were the primary providers of HS surgical care, plastic surgeons submitted more claims per physician. Plastic surgeons also performed the majority of procedures that involved a skin graft or rotation flap, with a previous study showing that ﻿flap reconstructions by plastic surgeons had significantly shorter operation times and lower transfusion rates than those by general surgeons, reflecting the specialized training plastic surgeons receive in reconstructive procedures [13]. Investigation into the education of relevant surgical programs on specialized HS surgical care may highlight areas of training requiring further improvement.

Furthermore, it is likely that more surgeons are choosing narrower scopes of practice, especially in general surgery where broader scopes of surgical services are diminishing with highly specialized postresidency fellowships [39]. This is reflected in the study, with more HS surgery claims being accompanied by more claims per provider but fewer providers overall. Increased specialization and narrowing scopes of practice may also lead to patients having to travel further to receive care from an available provider. Accordingly, approximately one-third of patients received care away from their home, with this number also increasing over time. This has important implications for postsurgical follow-up care, with HS being a chronic relapsing disease requiring months to even years of follow-up post-surgical excision [6,31]. This highlights the need for recruitment of more surgeons to perform HS surgery as well as the training of rural surgeons on the surgical treatment of HS.

### Strengths and Limitations

This study benefited from the use of a comprehensive, large, longitudinal database, allowing for future comparison studies. However, a limitation to this study was the lack of a specific diagnostic code for HS. Therefore, we were unable to evaluate and compare changes in HS surgery over time to changes in HS claims. Future studies should explore this association, to further help characterize patient access to HS surgical care. Second, the two billing codes used in this study, R059 and R060, do not reflect the entirety of procedures that can be offered for HS, such as abscess drainage, laser treatments, or electrosurgical peeling procedures [5,40]. However, these treatment modalities are relatively novel and are rarely performed as standard of care. Procedures such as abscess drainage also have high recurrence rates of up to 100% and are not performed as a curative option [5,41]. Furthermore, we were unable to assess demographic factors of patients and physicians such as gender, race, and ethnicity, limiting our analysis. However, evidence suggests unequal access leading to racial disparities in surgical care [42]. Further investigation into HS patient and physician demographic factors would be impactful to analyze in future studies.

### Comparison With Prior Work

Barriers to seeking HS care have previously been reported to include a lack of knowledge about HS among providers, difficulty accessing specialists, poor patient-physician communication, distrust in the medical community, and patients’ experiences with HS [43]. This can be amplified by the significant delay to diagnosis that adds to patient frustration and disease severity and affects the likelihood of receiving well-planned, individual management [35]. Despite ongoing research on new treatment modalities, there is also a need to evaluate the circumstances of these barriers including access to operating room time, extent of provider education on HS, role of subspecialization among surgical providers, and public awareness around HS. This also translates to potential areas for public health authorities and hospital administrations to improve HS care, specifically in regard to increasing operating room time for HS surgeries.

### Conclusions

Unfortunately, no significant improvements in patient access to surgery were seen across the study period, with annual HS claims rising overall, number of providers decreasing, and patients travelling further to access surgery. A lack of access to operating room time and narrowing scopes of practice may be contributing factors potentially worsening access over time. Further research on HS surgery, including evaluation into system barriers across the continuum of HS diagnosis and management, are required in order to improve access to surgical care for HS patients.

## Appendix

Multimedia Appendix 1 Supplemental Table 1. Claims submitted for hidradenitis suppurativa (HS) surgery by specialty.

Multimedia Appendix 2 Supplemental Figure 1. (a) Percentage of claims submitted by physician and patient location. (b) Percentage of claims billed by specialty using physician location.

Multimedia Appendix 3 Supplemental Figure 2. Percentage of claims submitted by physician and patient location (defined as their Local Health Integration Network [LHIN]) over time. Shown by claims submitted by physicians who resided in the same location as the patient at the time of surgery, and by those who did not.

## Abbreviations

HS: hidradenitis suppurativa
LHIN: Local Health Integration Network
OHIP: Ontario Health Insurance Plan
