# Supplementary material for: Exploring Access to Surgical Interventions for Hidradenitis Suppurativa: Retrospective Population-Based Analysis
Source: JMIR Dermatol. 2021 Dec 14;4(2):e31047. doi: 10.2196/31047 (PMC10334952; doi:10.2196/31047)
Supplement: Multimedia Appendix 1 [file derma_v4i2e31047_app1.docx]

**Supplemental Table 1.** Claims submitted for HS surgery by specialty.

| **Specialty Billed** | **No. (%) of Claims for R059** | **No. (%) of Claims for R060** |
| --- | --- | --- |
| General Surgery | 3,687 (61.6) | 247 (20.4) |
| Plastic Surgery | 2,179 (36.4) | 928 (76.6) |
| Obstetrics and Gynaecology | 57 (1.0) | 6 (0.5) |
| Urology | 48 (0.8) | 10 (0.8) |
| Dermatology | 13 (0.2) | 20 (1.7) |
