# Supplementary figures and images for: Exploring Access to Surgical Interventions for Hidradenitis Suppurativa: Retrospective Population-Based Analysis
Source: JMIR Dermatol. 2021 Dec 14;4(2):e31047. doi: 10.2196/31047 (PMC10334952; doi:10.2196/31047)

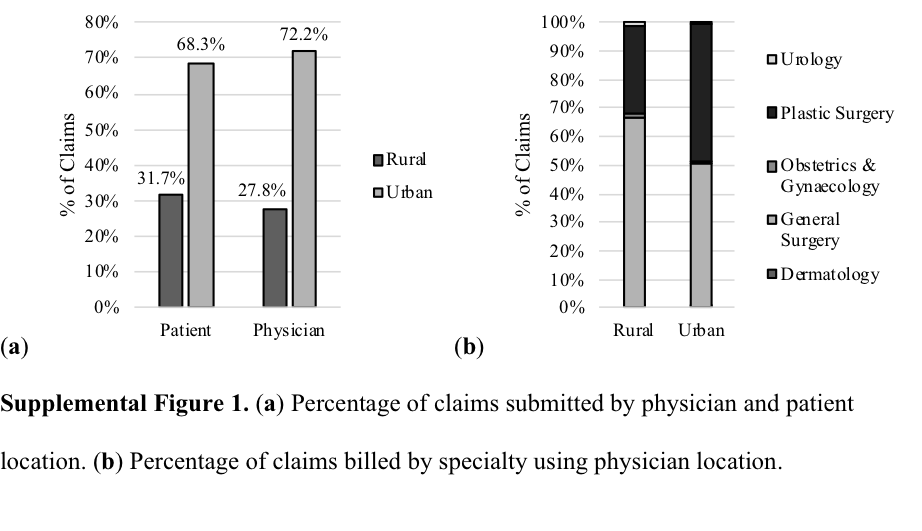

Supplement: Multimedia Appendix 2 [file derma_v4i2e31047_app2.png]

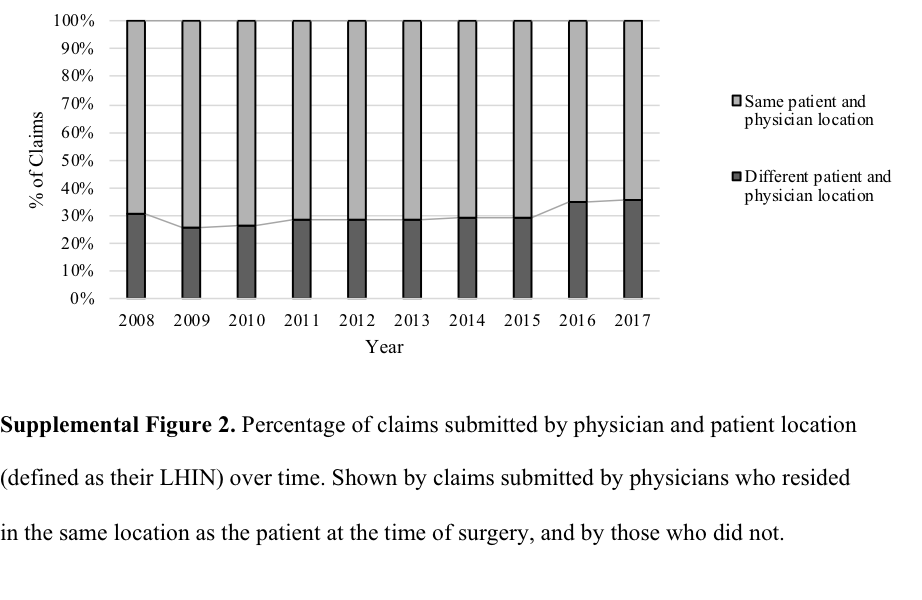

Supplement: Multimedia Appendix 3 [file derma_v4i2e31047_app3.png]
